# Tumor tissue levels of Tissue Inhibitor of Metalloproteinases-1 (TIMP-1) and outcome following adjuvant chemotherapy in premenopausal lymph node-positive breast cancer patients: A retrospective study

**DOI:** 10.1186/1471-2407-9-322

**Published:** 2009-09-10

**Authors:** Anne-Sofie Schrohl, Maxime P Look, Marion E Meijer-van Gelder, John A Foekens, Nils Brünner

**Affiliations:** 1 University of Copenhagen, Faculty of Life Sciences, Department of Veterinary Disease Biology, Ridebanevej 9, DK-1870 Frederiksberg C, Denmark; 2 Erasmus MC, Department of Medical Oncology, Josephine Nefkens Institute and Cancer Genomics Center, Dr. Molewaterplein 50, 3015 GE Rotterdam, the Netherlands

## Abstract

**Background:**

We have previously demonstrated that high tumor tissue levels of TIMP-1 are associated with no or limited clinical benefit from chemotherapy with CMF and anthracyclines in metastatic breast cancer patients. Here, we extend our investigations to the adjuvant setting studying outcome after adjuvant chemotherapy in premenopausal lymph node-positive patients. We hypothesize that TIMP-1 high tumors are less sensitive to chemotherapy and accordingly that high tumor tissue levels are associated with shorter survival.

**Methods:**

From our original retrospectively collected tumor samples we selected a group of 525 pre-menopausal lymph node-positive patients (adjuvant treatment: CMF, 324 patients; anthracycline-based, 99 patients; no adjuvant chemotherapy, 102 patients). TIMP-1 levels were measured using ELISA in cytosolic extracts of frozen primary tumors. TIMP-1 was analyzed as a continuous variable and as a dichotomized one using the median TIMP-1 concentration as a cut point between high and low TIMP-1 groups. We analyzed the benefit of adjuvant CMF and anthracyclines in univariate and multivariable survival models; endpoints were disease-free (DFS) and overall survival (OS).

**Results:**

In this selected cohort of high-risk patients, and in the subgroup of patients receiving no adjuvant therapy, TIMP-1 was not associated with prognosis. In the subgroup of patients treated with anthracyclines, when analyzed as a continuous variable we observed a tendency for increasing TIMP-1 levels to be associated with shorter DFS (multivariable analysis, HR 1.75, 95% CI 1.00-3.07, P = 0.05) and a significant association between increasing TIMP-1 and shorter OS in both univariate (HR 3.52, 95% CI 1.54-8.06, P = 0.003) and multivariable analyses (HR 4.19, 95% CI 1.67-10.51, P = 0.002). No statistically significant association between TIMP-1 and DFS was observed in the CMF-treated patients although high TIMP-1 was associated with shorter OS when analyzed as a dichotomized variable (HR 1.64, 95% CI 1.02-2.65, P = 0.04).

**Conclusion:**

In the subgroup of patients receiving adjuvant chemotherapy we found an association between shorter survival after treatment in TIMP-1 high patients compared with TIMP-1 low patients, especially in patients receiving anthracycline-based therapy. This suggests that high tumor tissue levels of TIMP-1 might be associated with reduced benefit from classical adjuvant chemotherapy. Our findings should be validated in larger prospective studies.

## Background

Many patients are offered adjuvant chemotherapy after surgery for primary breast cancer. Several options exist (reviewed in [1]) but currently, adjuvant chemotherapy only reduces breast cancer mortality by up to 50% [2] and hence further improvement is needed. Moreover, chemotherapy is most often associated with substantial side effects. In theory, by attacking the tumor cells effectively at an early stage unnecessary growth and potential selection of resistant clones is avoided and a better response to adjuvant treatment is to be expected. Choosing the most effective adjuvant chemotherapy regimen up front should thus lead to a further reduction in disease recurrences and, accordingly, to increased survival of the patients. In addition, avoiding ineffective treatments would reduce unneeded toxicity and burden for the patients and the substantial costs associated with administration of adjuvant chemotherapy.

Prescription of chemotherapy with no benefit to the patient could be limited through identification and use of predictive markers. Predictive markers serve as a tool for tailoring therapy for individual patients, however, the number of approved markers in primary breast cancer is limited [3]. Thus, additional markers are needed to improve planning of a more personalized adjuvant treatment.

TIMP-1 is a naturally occurring inhibitor belonging to the matrix metalloproteinase (MMP) system, and besides its MMP-inhibitory functions TIMP-1 also appears to independently influence cell growth and apoptosis (for reviews, see [4,5]). In metastatic breast cancer patients, we reported that a high level of TIMP-1 in the primary tumor tissue is associated with decreased objective response to chemotherapy [6], and a report showing a similar association in the adjuvant setting was recently presented [7]. In addition, it appears that TIMP-1 may be combined with *TOP2A *for prediction of response to anthracycline-based chemotherapy [8]. Similarly, high levels of plasma and serum TIMP-1 are associated with a decreased response to endocrine therapy in metastatic breast cancer patients [9,10]. Besides the predictive information, which can apparently be gained from TIMP-1, several publications demonstrate that high levels of TIMP-1 protein in breast cancer tissue [11-14], plasma and serum [12,15-17] are associated with a poor prognosis. Based on these previous studies, it may be concluded that TIMP-1 appears to carry predictive as well as prognostic information; it has not been clarified, though, whether TIMP-1 carries mainly prognostic or predictive information and whether this information may be restricted to certain patient subgroups.

To further study the possible use of TIMP-1 as a biomarker in breast cancer patients, the present study investigates the association between levels of TIMP-1 in cytosolic tumor tissue extracts and outcome following adjuvant chemotherapy with cyclophosphamide-methotrexate-5-fluorouracil (CMF) or an anthracycline-containing regimen (cyclophosphamide and 5-fluorouracil with adriamycin/epirubicin (CAF/CEF); or single-agent adriamycin (A)), or following no adjuvant treatment. We hypothesized that high levels of TIMP-1 are associated with decreased benefit from adjuvant chemotherapy and we investigated this hypothesis in a cohort of 525 premenopausal lymph node-positive breast cancer patients, who constitute a subgroup of a large cohort previously described in several studies [14,18,19]. First, we evaluated the association with prognosis in all patients included. The included subgroup of patients receiving no adjuvant therapy allowed for evaluation of association between TIMP-1 and outcome without any influence from adjuvant therapy; by definition, this is evaluation of prognosis. Secondly, in patients treated with adjuvant chemotherapy we analyzed the predictive impact of TIMP-1. The endpoints evaluated in the present study are DFS and OS. In particular, for the analyses of predictive impact of TIMP-1, DFS is considered the most informative as this endpoint is not influenced by subsequent systemic therapy. The Reporting Recommendations for Tumor Marker Prognostic Studies (REMARK [20]) were adhered to where ever applicable.

## Methods

### Patients and study design

Tumor tissue extracts included in the present study were collected between 1979 and 1994. Our protocol for studying molecular markers associated with disease progression was approved by the institutional Medical Ethics Committee of the Erasmus MC Rotterdam (MEC no. 02.953). The present study, in which coded tissues were used, was performed in accordance with the Code of Conduct of the Federation of Medical Scientific Societies in the Netherlands [21].

Patients were selected for the present study from a cohort of 2984 breast cancer patients [14] for which the original inclusion/exclusion criteria are described in [18]. Here, only patients who had lymph node-positive breast cancer and who were premenopausal at the time of diagnosis were included. This is due to the fact that very few postmenopausal and no lymph node-negative patients received adjuvant chemotherapy at the time when these samples were collected. Furthermore, only patients who received adjuvant chemotherapy with CMF, CEF, CAF or single-agent A, or who received no adjuvant chemo- or endocrine therapy were included. Treatment decisions were based on standard rules at the time. Finally, patients were included in the present study based on the availability of information about TIMP-1 concentration in the primary tissue, originally measured in stored cytosolic extracts after estrogen and progesterone receptor (ER/PgR) determination and used for a previous study [14].

A total of 525 patients fulfilled the inclusion criteria; 164 of these were ≤ 40 years, and 361 were 41-55 years. Among included patients, 292 patients had 1-3 positive lymph nodes and 233 had more than 3 tumor-positive axillary lymph nodes. In 175 patients, tumors were ≤ 2 cm (pT_1_), 270 patients had tumors larger than 2 cm and ≤ 5 cm (pT_2_), and 80 patients had tumors larger than 5 cm or skin or chest wall involvement (pT_3+4_). Differentiation grade was well or moderate in 103 tumors, poor in 291 tumors and unknown in 131 tumors. ER/PgR positive tumors were present in 432 patients. All patients had surgical removal of their tumor (313 mastectomies, 212 lumpectomies) and 391 patients received adjuvant radiotherapy (RT; of the chest wall and/or local or regional lymph nodes). A total of 423 patients received adjuvant chemotherapy; 324 had CMF, 99 had an anthracycline-containing regimen, and 102 patients had no adjuvant systemic therapy. The characteristics of the total patient group as well as subgroups according to adjuvant treatment are summarized in Table 1. In the table, and in the following parts, the term "Untreated patients" refers to patients who received no adjuvant therapy; however, it should be emphasized that these patients were treated with surgery and some also with adjuvant radiotherapy.

**Table 1:** Patient and tumor characteristics and comparisons of patient groups

Characteristic	All patients N (%)	CMF-treated patients N (%)	Anthracycline-treated patients N (%)	Untreated patients N (%)	P^1^
**Age**					
≤ 40 years	164 (31)	110 (34)	28 (28)	26 (25)	0.21^2^
41-55 years	361 (69)	214 (66)	71 (72)	76 (75)	

**Involved lymph nodes**					
1-3	292 (56)	217 (67)	47 (47)	28 (27)	<0.001^2^
>3	233 (44)	107 (33)	52 (53)	74 (73)	

**Steroid hormone rec. status**					
Positive	432 (82)	269 (83)	76 (77)	87 (85)	0.24^2^
Negative	93 (18)	55 (17)	23 (23)	15 (15)	

**Tumor size**					
pT_1_	175 (33)	131 (40)	21 (21)	23 (23)	<0.001^2^
pT_2_	270 (51)	147 (45)	58 (59)	65 (64)	
pT_3+4_	80 (15)	46 (14)	20 (20)	14 (14)	

**Grade**					
Well/moderate	103 (20)	65 (20)	21 (21)	17 (17)	0.33^2^
Poor	291 (55)	174 (54)	61 (62)	56 (55)	
Unknown	131 (25)	85 (26)	17 (17)	29 (28)	

**Primary treatment**					
Lumpectomy	212 (40)	169 (52)	4 (4)	39 (38)	<0.001^2^
Mastectomy	313 (60)	155 (48)	95 (96)	63 (62)	

**Events^3^**					
Recurrences (disease failure)	268	140	58	70	
Deaths	149	75	32	42	

**TIMP-1 (median, range)**	12.5 (0- 113)	12.0 (0- 106)	13.5 (0-51.2)	13.8 (0- 113)	0.20^4^

All patients, N = 525; CMF-treated patients, N = 324; anthracycline-treated, N = 99; untreated patients, N = 102.

^1^Testing the hypothesis that subgroups (CMF- or anthracycline treated and untreated) are similar

^2^Pearson X^2 ^test

^3^Events when censored at 60 months

^4^Kruskal-Wallis equality-of-populations rank test

Follow-up consisted of routine examinations every 3-6 months during the first 5 years and once a year thereafter. The median survival time of patients alive was 99 (range, 9-255) months (CMF-treated patients, 103 (range, 12-248) months; anthracycline-treated patients, 90 (range, 9-213) months; untreated patients, 93 (range, 15-255) months).

Median survival of patients alive represents median observation time.

### Assays and tumor specimens

TIMP-1 concentrations were originally determined for use in a large study of the association between TIMP-1 and prognosis, and this study also included validation of the assay with the present extraction buffer [14].

In brief, tumor tissue specimens were prepared following instructions from the European Organisation for Research and Treatment of Cancer (EORTC) regarding extraction of tumor tissue for determination of cytosolic ER and PgR [22]. Levels of TIMP-1 in the cytosols were measured using an established, validated in-house enzyme-linked immunosorbent assay (ELISA) [23]. Concentrations of total protein were measured using the Coomassie Brilliant Blue Method (Bio-Rad Laboratories, Hercules, CA). ER and PgR levels were assessed previously by ligand-binding assay or enzyme immunoassay as described in [24] and the cut point used for classification as ER/PgR positive or negative was 10 fmol/mg of protein.

### Data analysis and statistics

Differences in TIMP-1 levels were assessed with the Mann-Whitney U test or the Kruskal-Wallis equality-of-populations rank test when appropriate. In these tests, patient and tumor characteristics were used as grouping variables. Associations between continuous variables were tested with the Spearman rank correlation (r_s_). Differences between treatment groups were tested with the Pearson X^2 ^test. Cox proportional hazard models were applied to compute the hazard ratio (HR). Disease-free survival (DFS) and overall survival (OS) were used as endpoints. For DFS any relapse or secondary breast cancer was counted as failure; non-failing patients were censored at last date of contact. For OS, death of any cause was counted as a failure. Surviving patients were censored at last day of follow-up.

The protocols for adjuvant treatment (RT on the axilla and/or systemic therapy), depending on the number of tumor-positive lymph nodes (1-3, >3), were changed during the study period. To accommodate these changes we used a combination of nodal status (1-3, >3) and RT on the axilla (no, yes) to stratify all analyses.

Proportional hazards assumptions were tested based on Schoenfeld residuals. As to be expected, the proportional hazards assumption was violated for hormone receptor status and accordingly, all analyses were stratified for hormone receptor status (negative, positive). Hence, none of the variables used for stratification (nodal status, RT, hormone receptor status) were included in the multivariable analyses. Age, tumor size, and malignancy grade defined the base model to correct for classical prognostic factors. In multivariable analyses TIMP-1 was added to this model. In none of these analyses the proportional hazards assumption was violated.

To reduce skewness of the distribution of TIMP-1 levels, these were log-transformed. No interactions were observed between TIMP-1 and the classical prognostic factors or adjuvant therapy in the analysis for DFS. The interaction analysis investigates whether the contribution to the survival model of the one variable is dependent upon the values of the other; the estimates for the survival model will be different for the subgroups if there is an interaction [25].

TIMP-1 levels were dichotomized by the overall median (12.5 ng/mg of total protein). Dichotomized levels were used for the survival curves using the method of Kaplan and Meier.

In this dataset, three groups relating to systemic adjuvant treatment were available: patients treated with CMF, patients treated with anthracyclines, and patients who received no adjuvant chemotherapy. In the analyses of all patients we used the group receiving no adjuvant chemotherapy as the reference group. For both endpoints (DFS, OS), the hypothesis that these untreated patients are not different, regardless of tumor tissue TIMP-1 levels (low, high), was maintained.

**Figure 1:**
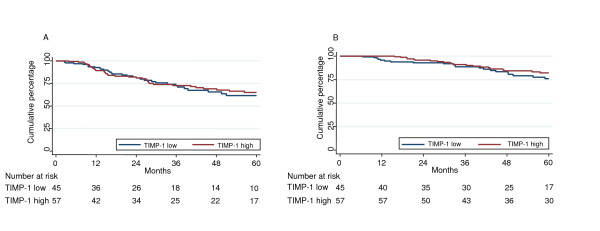
**Kaplan-Meier plot showing the DFS (A) and OS (B) of untreated TIMP-1 low and high patients**. The median TIMP-1 concentration of the total patient group (12.5 ng/mg of total protein) was used as cut point (TIMP-1 low patients N = 57, TIMP-1 high patients N = 45). Cox univariate regression analysis; DFS: HR 0.93, 95% CI 0.58-1.49, P = 0.76; OS: HR 0.72, 95% CI 0.39-1.33, P = 0.30.

**Figure 2:**
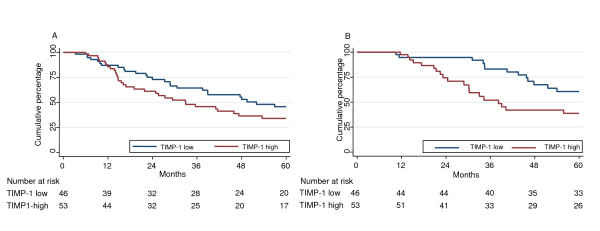
**Kaplan-Meier plot showing the DFS (A) and OS (B) of TIMP-1 low and high anthracycline-treated patients**. The median TIMP-1 concentration of the total patient group (12.5 ng/mg of total protein) was used as cut point (TIMP-1 low patients N = 46, TIMP-1 high patients N = 53). Cox univariate regression analysis; DFS: HR 1.52, 95% CI 0.88-2.63, P = 0.13; OS: HR 2.53, 95% CI 1.19-5.39, P = 0.02.

**Figure 3:**
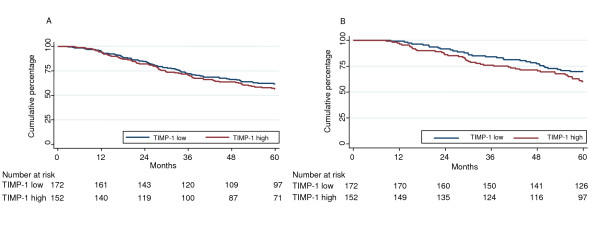
**Kaplan-Meier plot showing the DFS (A) and OS (B) of TIMP-1 low and high CMF-treated patients**. The median TIMP-1 concentration of the total patient group (12.5 ng/mg of total protein) was used as cut point (TIMP-1 low patients N = 172, TIMP-1 high patients N = 152). Cox univariate regression analysis; DFS: HR 1.15, 95% CI 0.82-0.1.61, P = 0.42; OS: HR, 1.44 (0.91-2.29), P = 0.12.

For all analyses, the survival times were censored at 60 months because the numbers of patients at risk in some treatment groups were low at 5 years; the numbers of patients at risk in the treatment subgroups appear from the figures (Figure 1, 2 and 3).

Computations were performed with the STATA statistical package, version 10.1 (STATA Corp., College Station, TX). All P-values are two-sided and P < 0.05 was considered statistically significant.

## Results

### Characterization and comparison of patient subgroups

Patients were subdivided according to adjuvant chemotherapy regimen received (CMF, 324 patients; anthracycline-containing, 99 patients; no adjuvant chemotherapy, 102 patients). Table 1 summarizes the characteristics of the 3 subgroups together with the characteristics of the total patient group.

The three subgroups differed significantly with respect to the number of tumor-positive lymph nodes, tumor size, and primary treatment (Pearson X^2 ^test, P < 0.001, Table 1). Thus, patients who received CMF more often had only 1-3 involved lymph nodes and also had smaller tumors (pT_1_) when compared with untreated patients and with patients who received anthracycline-based chemotherapy. Looking at primary treatments, more patients had a lumpectomy among CMF-treated and patients receiving no adjuvant chemotherapy than among anthracycline-treated patients. In addition to this, fewer anthracycline-treated patients received adjuvant RT. The differences in number of involved lymph nodes and adjuvant RT were partly accounted for in the stratification.

### TIMP-1 levels and association of TIMP-1 with clinicopathological variables

The median TIMP-1 concentration in the tissue extracts was 12.5 ng/mg of total protein (range, 0-113 ng/mg protein). Median tumor tissue TIMP-1 levels in subgroups according to adjuvant treatment were as follows: In CMF-treated patients 12.0 ng/mg of total protein (range, 0-106 ng/mg); in patients who received an anthracycline-containing regimen 13.5 ng/mg protein (range, 0-51.2 ng/mg); and in patients who received no adjuvant chemotherapy 13.8 ng/mg protein (range, 0-113 ng/mg). These levels were not significantly different (Kruskal-Wallis equality-of-populations rank test, P = 0.20, Table 1).

Analyzing the total group of patients, TIMP-1 was significantly associated with age as younger patients had significantly lower levels of TIMP-1 in their tumors (Spearman rank correlation analysis, r_s _= 0.10, P = 0.02). No significant association was found between TIMP-1 and number of tumor-positive lymph nodes, steroid hormone receptor status, tumor size, grade, adjuvant RT, or type of surgery.

When subgroups according to adjuvant treatment were analyzed separately we found no significant associations between TIMP-1 and age, number of tumor-positive lymph nodes, tumor size, grade, adjuvant RT, or type of surgery in any of the subgroups. In the subgroup of CMF-treated patients, however, we found an association between TIMP-1 and hormone receptor status with higher tumor tissue TIMP-1 levels in patients with hormone receptor negative tumors (Mann-Whitney test, P = 0.04).

### TIMP-1 and prognosis

The prognostic impact of TIMP-1 was analyzed in the total patient group and in further detail in the group of patients who received no adjuvant chemotherapy. In these analyses, TIMP-1 was analyzed both as a continuous log-transformed variable and as a dichotomized variable with patients divided into two groups of high and low tumor tissue TIMP-1 levels, respectively, by the median TIMP-1 concentration of the total patient group (12.5 ng/mg protein). As described in the Statistics part, all analyses were censored at 60 months and stratified for the number of involved lymph nodes, RT of the axilla, and hormone receptor status.

In Cox univariate regression analysis including all 525 patients, increasing tumor tissue TIMP-1 concentrations were not significantly associated with DFS or OS neither when analyzed as a continuous log-transformed variable (DFS: HR 1.15, 95% CI 0.93-1.42, P = 0.20; OS: HR 1.17, 95% CI 0.88-1.57, P = 0.28) nor as a dichotomized variable (DFS: HR 1.13, 95% CI 0.88-1.43, P = 0.34; OS: HR 1.24, 95% CI 0.89-1.71, P = 0.20).

We then analyzed the subgroup of patients receiving no adjuvant chemotherapy separately as the outcome of this group is uninfluenced by systemic treatment. In both univariate and multivariable survival analyses of this subgroup we found no association between TIMP-1 and outcome (DFS, OS) neither when including TIMP-1 as a continuous variable nor as a dichotomized variable (Table 2). The DFS and OS of TIMP-1 high and low untreated patients are illustrated in Figure 1A and 1B, respectively, which show that the curves describing the two groups are super imposable.

**Table 2:** Univariate and Multivariable survival analyses of the subgroup of untreated patients, N = 102*

Univariate		DFS		OS	
		
		HR (95% CI)	P	HR (95% CI)	P
**TIMP-1**	Continuous variable	0.96 (0.63-1.45)	0.84	0.72 (0.39-1.33)	0.29
	
	High *vs*. low	0.93 (0.58-1.49)	0.76	0.72 (0.39-1.33)	0.3

**Multivariable**		**DFS**		**OS**	
		**HR (95% CI)**	**P**	**HR (95% CI)**	**P**

**TIMP-1**	Continuous variable	0.95 (0.60-1.51)	0.84	0.80 (0.41-1.57)	0.52
	
	High *vs*. low	0.95 (0.56-1.62)	0.86	0.80 (0.41-1.54)	0.5

**Age**	41-55 years *vs*. ≤ 40 years	0.53 (0.31-0.89)	0.02	0.40 (0.21-0.77)	0.01

**Tumor size **	Stage 2 (> 2 cm) *vs*. stage 1 (≤ 2 cm)	1.76 (0.93-3.33)	0.19	1.77 (0.72-4.36)	0.37
				
	Stage 3 (>5 cm or chest wall/skin involvement) *vs*. stage 1 (≤ 2 cm)	1.35 (0.58-3.14)		2.01 (0.62-6.45)	

**Grade **	Unknown *vs*. poor	0.70 (0.40-1.22)	0.39	0.83 (0.41-1.67)	0.51
				
	Well/moderate *vs*. poor	0.74 (0.33-1.64)		0.54 (0.18-1.64)	

* Analyses stratified for hormone receptor status, nodal status and RT on the axilla. The results for age, tumor size and grade are from the base model not including TIMP-1. TIMP-1 was added separately as a continuous variable and then as a dichotomized one. The coefficients for age, tumor size and grade are similar with TIMP-1 included.

There were no significant differences among untreated TIMP-1 high and low patients with respect to clinicopathological parameters (type of surgery, age, number of tumor-positive lymph nodes, tumor size, hormone receptor status, tumor grade, RT; data not shown).

### TIMP-1 and prediction

To address the question of a possible predictive impact of TIMP-1 we analyzed the outcome (DFS, OS) of patients who had received adjuvant systemic therapy. We used two different approaches to address this question; both approaches were considered justified since there appears to be no, or a modest, association between TIMP-1 and prognosis in the patients studied here. Consequently, the reported differences are not due to a combined prognostic and predictive impact of TIMP-1 but rather a predominantly predictive one. In our first analysis, we evaluated the benefit from treatment in TIMP-1 high and low patients by comparing with the outcome of patients who did not receive systemic adjuvant therapy, and secondly we compared the outcome of TIMP-1 high- and low patients within treatment subgroups.

#### Comparison with patients who did not receive adjuvant chemotherapy

First, we compared DFS of patients treated with adjuvant anthracycline-containing chemotherapy or adjuvant CMF with DFS of untreated patients. For this purpose, a variable combining TIMP-1 status and adjuvant chemotherapy was created: Patients who received adjuvant chemotherapy were divided into TIMP-1 high and low groups, again applying the median TIMP-1 concentration of the total patient group as a cut point. Patients who did not receive systemic adjuvant therapy were included as one group (high and low TIMP-1) for reference purposes.

First, we performed Cox univariate regression analysis using the untreated patients as a reference. Among anthracycline-treated patients neither TIMP-1 low nor TIMP-1 high patients had a significantly improved survival when using the untreated patients as a reference group (TIMP-1 low patients: HR 0.74, 95% CI 0.45-1.24, P = 0.26; TIMP-1 high patients: HR 1.03, 95% CI 0.63-1.67, P = 0.92). When comparing the TIMP-1 low and high CMF-treated groups with the untreated patients group, we found that both TIMP-1 low and high groups had a significantly better DFS than untreated patients (TIMP-1 low patients: HR 0.51, 95% CI 0.36-0.73, P < 0.001; TIMP-1 high patients: HR 0.58, 95% CI 0.41-0.81, P = 0.002).

We also analyzed the benefit from adjuvant treatment, when compared with untreated patients, in a multivariable model. As mentioned our base model included age, tumor size, malignancy grade, and adjuvant chemotherapy and TIMP-1 was added to this model. Among patients treated with anthracycline-containing chemotherapy a trend towards a better outcome in TIMP-1 low patients compared with untreated patients was observed although neither TIMP-1 low nor TIMP-1 high patients had a significantly better DFS than untreated patients (TIMP-1 low patients: HR 0.71, 95% CI 0.42-1.19, P = 0.19; TIMP-1 high patients: HR 0.97, 95% CI 0.60-1.57, P = 0.90). In the CMF-treated subgroup both TIMP-1 low and high patients had a significantly longer DFS than untreated patients (TIMP-1 low patients: HR 0.48, 95% CI 0.33-0.69, P < 0.001; TIMP-1 high patients: HR 0.57, 95% CI 0.41-0.81, P = 0.001). In this multivariable model younger age and increase in tumor size were associated with a significantly shorter DFS. The results of the multivariable analysis combining TIMP-1 status and adjuvant therapy are shown in Table 3.

**Table 3:** Multivariable analysis of DFS after adjuvant chemotherapy with CMF or anthracyclines *vs*. no adjuvant treatment*

		HR (95% CI)	P
**Anthracycline-treatment**	TIMP-1 low *vs*. all untreated	0.71 (0.42-1.19)	0.19
	
	TIMP-1 high *vs*. all untreated	0.97 (0.60-1.57)	0.90

**CMF-treatment**	TIMP-1 low *vs*. all untreated	0.48 (0.33-0.69)	<0.001
	
	TIMP-1 high *vs*. all untreated	0.57 (0.41-0.81)	0.001

**Age**	41-55 years *vs*. ≤ 40 years	0.63 (0.49-0.81)	<0.001

**Tumor size**	Stage 2 (> 2 cm) *vs*. stage 1 (≤ 2 cm)	1.80 (1.33-2.45)	<0.001
		
	Stage 3 (>5 cm or chest wall/skin involvement) *vs*. stage 1 (≤ 2 cm)	1.86 (1.26-2.74)	

**Grade**	Unknown *vs*. poor	0.83 (0.61-1.11)	0.08
		
	Well/moderate *vs*. poor	0.69 (0.49-0.98)	

* Analyses stratified for hormone receptor status, nodal status and RT on the axilla. All patients N = 525: anthracycline-treated low TIMP-1, 46 patients; anthracycline-treated high TIMP-1, 53 patients; CMF-treated low TIMP-1, 172 patients; CMF-treated high TIMP-1, 152 patients; untreated (high and low TIMP-1, combined), 102 patients.

In a similar multivariable analysis of OS, a more pronounced difference was found in benefit from anthracycline-based therapy in TIMP-1 low and high patients, when compared with untreated patients (TIMP-1 low patients: HR 0.54, 95% CI 0.26-1.11, P = 0.094; TIMP-1 high patients: HR 1.20, 95% CI 0.64-2.26, P = 0.58); none of the groups, though, had a significantly better outcome than patients who received no adjuvant chemotherapy. Among CMF-treated patients, both TIMP-1 low and high patients had a significantly better OS than untreated patients (TIMP-1 low patients: HR 0.42, 95% CI 0.26-0.70), P = 0.001; TIMP-1 high patients: HR 0.64, 95% CI 0.41-1.00, P = 0.05). Also in this multivariable model, age and tumor size were significantly associated with survival.

#### Comparison of TIMP-1 low- and high patients within treatment subgroups

We then compared the outcome after therapy and the association with increasing tumor tissue TIMP-1 separately within each treatment subgroup (anthracycline-treated, CMF). We performed analyses including TIMP-1 both as a continuous log-transformed variable and as a dichotomized variable with patients divided into two groups of high and low tumor tissue TIMP-1 levels, respectively, by the median TIMP-1 concentration of the total patient group.

In Cox univariate regression analyses, both in patients who received adjuvant anthracycline-based chemotherapy and in CMF-treated patients, we found no statistically significant associations between TIMP-1 and DFS (Table 4, Figures 2A and 3A); however, in anthracycline-treated patients, despite the non-significant results, there was a trend for TIMP-1 high patients to have a worse outcome than TIMP-1 low patients, especially when analyzing TIMP-1 as a continuous variable (HR 1.66, 95% CI 0.96-2.85, P = 0.07). Additionally, in the anthracycline-treated group high TIMP-1 was associated with a significantly shorter OS both when analyzed as a continuous variable (HR 3.52, 95% CI 1.54-8.06, P = 0.003) and as a dichotomized one (HR 2.53, 95% CI 1.19-5.39, P = 0.02) whereas no differences were observed among TIMP-1 high and low patients in the CMF-treated subgroup (Table 4, Figures 2B and 3B).

**Table 4:** Univariate survival analysis of anthracycline- and CMF-treated subgroups, N = 99 and N = 324*

		DFS		OS	
		
		HR (95% CI)	P	HR (95% CI)	P
**Anthracycline-treated patients**	TIMP-1 (continuous variable)	1.66 (0.96-2.85)	0.07	3.52 (1.54-8.06)	0.003
	
	TIMP-1 (high *vs*. low)	1.52 (0.88-2.63)	0.13	2.53 (1.19-5.39)	0.02

**CMF-treated patients**	TIMP-1 (continuous variable)	1.12 (0.84-1.51)	0.44	1.22 (0.82-1.81)	0.32
	
	TIMP-1 (high *vs*. low)	1.15 (0.82-1.61)	0.42	1.44 (0.91-2.29)	0.12

* Analyses stratified for hormone receptor status, nodal status and RT on the axilla.

We then performed separate multivariable survival analyses in anthracycline-treated and in CMF-treated patients, respectively (Table 5 and 6). As before, TIMP-1 was added to the base model first as a continuous and then as a dichotomized variable. In the analysis of the anthracycline-treated subgroup, TIMP-1 analyzed as a continuous variable showed a strong tendency for higher levels to be associated with a shorter DFS (HR 1.75, 95% CI 1.00-3.07, P = 0.05). In patients who received adjuvant CMF we found no association between TIMP-1 and DFS; in this model younger age and increase in tumor size were associated with a shorter DFS. When including TIMP-1 in the multivariable models, the coefficients for the other variables included were similar. In addition, among patients treated with adjuvant anthracycline-based therapy we found a highly significant association with OS both when included as a continuous (HR 4.19, 95% CI 1.67-10.51, P = 0.002) and as a dichotomized variable (HR 2.59, 95% CI 1.14-5.88, P = 0.02). In CMF-treated patients, TIMP-1 was associated with shorter OS only when analyzed as a dichotomized variable (HR = 1.64, 95% CI 1.02-2.65, P = 0.04).

**Table 5:** Multivariable analyses of anthracycline-treated patients, N = 99*

		DFS		OS	
		
		HR (95% CI)	P	HR (95% CI)	P
**TIMP-1**	Continuous variable	1.75 (1.00-3.07)	0.05	4.19 (1.67-10.51)	0.002
	
	High *vs*. low	1.48 (0.84-2.61)	0.17	2.59 (1.14-5.88)	0.02

**Age**	41-55 years *vs*. ≤ 40 years	0.68 (0.37-1.25)	0.22	0.80 (0.37-1.72)	0.57

**Tumor size**	Stage 2 (> 2 cm) *vs*. stage 1 (≤ 2 cm)	2.13 (0.91-4.98)	0.13	3.63 (0.82-16.13)	0.14
				
	Stage 3 (>5 cm or chest wall/skin involvement) *vs*. stage 1 (≤ 2 cm)	2.31 (0.87-6.11)		2.77 (0.54-14.14)	

**Grade**	Unknown *vs*. poor	0.90 (0.43-1.92)	0.88	0.16 (0.02-1.21)	0.06
				
	Well/moderate *vs*. poor	0.82 (0.35-1.88)		1.11 (0.39-3.15)	

* Analyses stratified for hormone receptor status, nodal status and RT on the axilla. The results for age, tumor size and grade are from the base model not including TIMP-1. TIMP-1 was added separately as a continuous variable and then as a dichotomized one. The coefficients for age, tumor size and grade were similar with TIMP-1 included.

**Table 6:** Multivariable analysis of CMF-treated patients, N = 324*

		DFS		OS	
		
		HR (95% CI)	P	HR (95% CI)	P
**TIMP-1**	Continuous variable	1.18 (0.87-1.59)	0.28	1.29 (0.87-1.91)	0.20
	
	High *vs*. low	1.23 (0.87-1.73)	0.25	1.64 (1.02-2.65)	0.04

**Age**	41-55 years *vs*. ≤ 40 years	0.60 (0.43-0.85)	0.004	0.53 (0.33-0.84)	0.007

**Tumor size**	Stage 2 (> 2 cm) *vs*. stage 1 (≤ 2 cm)	1.59 (1.07-2.38)	0.02	2.34 (1.31-4.20)	0.01
				
	Stage 3 (>5 cm or chest wall/skin involvement) *vs*. stage 1 (≤ 2 cm)	1.92 (1.15-3.22)		1.97 (0.92-4.20)	

**Grade**	Unknown *vs*. poor	0.83 (0.55-1.25)	0.24	1.05 (0.60-1.84)	0.62
				
	Well/moderate *vs*. poor	0.68 (0.43-1.10)		0.74 (0.38-1.46)	

* Analyses stratified for hormone receptor status, nodal status and RT on the axilla. The results for age, tumor size and grade are from the base model not including TIMP-1. TIMP-1 was added separately as a continuous variable and then as a dichotomized one. The coefficients for age, tumor size and grade were similar with TIMP-1 included.

Among anthracycline-treated patients there were no significant differences in clinicopathological characteristics between TIMP-1 high and low patients; among CMF-treated patients there were significantly more patients younger than 40 years in the TIMP-1 low subgroup and significantly more patients with more than 3 tumor-positive lymph nodes in the TIMP-1 high subgroup (data not shown).

#### Interactions

There were no statistically significant interactions between TIMP-1 and adjuvant chemotherapy in analyses of DFS. However, in analyses of OS there was a statistically significant interaction between TIMP-1 and anthracycline treatment both in a model including only adjuvant therapy and TIMP-1 (HR 3.60, 95% CI 1.38-9.38, P = 0.009) and in the model including age, tumor size and malignancy grade (HR 3.20, 95% CI 1.26-8.12, P = 0.014).

## Discussion

We recently reported that metastatic breast cancer patients with high tumor tissue levels of TIMP-1 had no benefit from chemotherapy with CMF and anthracycline-containing regimens suggesting that tissue TIMP-1 may be a predictive marker for response to chemotherapy [6], and data from studies of adjuvant treatment are now emerging [7,8]. TIMP-1 in plasma and serum has been suggested as a marker of prognosis [12,15-17] and additionally, high blood levels of TIMP-1 have been shown to predict resistance to endocrine therapy in patients with metastatic breast cancer [9,10]. It is still unclear, though, exactly how TIMP-1 is related with prognosis and prediction, and whether different information may be obtained from TIMP-1 in different patient subgroups.

In this study, we analyzed the relation between TIMP-1 and prognosis by evaluating outcome (DFS, OS) in the total group of 525 patients and in a subgroup of patients who had not received adjuvant chemotherapy and unexpectedly found no significant associations. The DFS analysis of the untreated group is particularly interesting as this analysis describes only the prognostic impact of TIMP-1 with no interference from systemic treatment; currently, this is rarely feasible as most patients, certainly among lymph node-positive patients, receive systemic adjuvant treatment. We previously reported a prognostic impact of TIMP-1 in our large cohort [14] and we expected to confirm this finding in the presently analyzed premenopausal untreated patients, who were also included in the original cohort. However, in the original study many lymph node-positive patients, in particular premenopausal patients, had received adjuvant systemic treatment and this may potentially have affected the analysis of prognostic impact. Thus, even if the presently analyzed subgroups are small, which makes it difficult to detect a modest association with prognosis, the apparent lack of prognostic impact in the present study suggests that the association of TIMP-1 with prognosis is limited to, or more pronounced in, other subgroups of patients.

We then addressed the question of a possible predictive impact of TIMP-1. Looking at anthracycline-treated patients, a comparison with patients who did not receive adjuvant chemotherapy (Table 3) showed that neither TIMP-1 low nor TIMP-1 high patients treated with anthracycline-based adjuvant therapy had a significantly better DFS than untreated patients. Thus, treatment with anthracycline-based chemotherapy appears to be associated with a very small benefit in both TIMP-1 high and low patients but it should be kept in mind that the anthracycline-treated subgroup is characterized by very poor prognostic features (larger tumors, many tumor-involved lymph nodes, Table 1). Moreover, looking closely at the HRs and CIs of the anthracycline-treated TIMP-1 low and high groups, TIMP-1 high patients appear to benefit even less than TIMP-1 low patients from the chemotherapy, compared with patients who received no adjuvant chemotherapy. This difference between TIMP-1 low and high patients was even more pronounced in the analysis of OS. In addition, in the subgroup analyses of anthracycline-treated patients (Tables 4 and 5) we observed a strong tendency for increasing TIMP-1 levels (continuous variable) to be associated with shorter DFS, and in the analyses of OS increasing TIMP-1 was strongly correlated with shorter survival in both univariate and multivariable analyses. We speculate that the differences seen in the subgroup analyses are mainly due to a predictive impact of TIMP-1 since there is a limited prognostic impact of TIMP-1 in this cohort. The interaction analysis which showed a statistically significant association between TIMP-1 and anthracycline therapy in the analyses of OS further suggests that high levels of tumor tissue TIMP-1 are predictive of reduced benefit from adjuvant anthracycline-based therapy.

Similarly, the benefit from CMF was first analyzed in the combined model with patients who received no adjuvant therapy as a reference group (Table 3). This analysis showed that both TIMP-1 high and TIMP-1 low patients had a significantly longer survival than untreated patients. In subgroup analyses of CMF-treated patients the only significant association between TIMP-1 and outcome was a modest association between TIMP-1 and OS in the multivariable analysis including TIMP-1 as dichotomized variable (Tables 4 and 6). Thus, in this cohort the benefit from adjuvant CMF appears to be similar in TIMP-1 high and low patients.

The endpoint of DFS is uninfluenced by therapy given in the metastatic setting; yet, the results in the present analyses of OS are stronger in terms of showing statistically significant associations, including a significant interaction between anthracycline treatment and TIMP-1. Since TIMP-1 is also associated with response to therapy in the metastatic setting [6] it could be speculated that this influences the analyses of OS in the present study. Thus, the statistically significant association in the analyses of OS illustrates a combination of predictive impact in the adjuvant as well as in the metastatic setting.

Several of the analyses presented show tendencies rather than statistically significant results and a number of aspects related to the patient cohort and the analyses should be mentioned. Firstly, all survival analyses were performed first including TIMP-1 as a continuous variable and then as a dichotomized one employing the median TIMP-1 concentration as a cut point. However, inclusion of TIMP-1 as a continuous variable, in particular in analyses of patients treated with anthracycline-based therapy, showed a stronger association between TIMP-1 and outcome than those employing the dichotomized variable. Thus, it could be speculated that the median TIMP-1 concentration is not an ideal cut point for separating TIMP-1 low and high patients, however, the limited number of patients did not allow for analyses employing more than one cut point.

In addition, the subgroups of patients who received no adjuvant chemotherapy or anthracycline-based therapy are small, and the anthracycline-treated group is characterized by having a very poor prognosis based on classical prognostic parameters. Moreover, the patient cohort included in the present study consists of premenopausal lymph node-positive patients who had left-over tumor extracts stored in the tumor bank. Thus, the cohort does not represent a consecutively and prospectively collected cohort but rather a selected one. The diversity of the patient cohort did complicate the analyses; there were substantial differences among subgroups with regard to primary treatment (surgery, RT), tumor size, degree of lymph node involvement, and patients were following different treatment protocols employed at different times. The stratified multivariable models account for the differences to some extent, but the diversity still should be kept in mind when interpreting the results. Yet, the use of archival samples dating as far back as 1979 allowed for inclusion of an untreated patient group and this offered a unique opportunity for evaluation of prognosis of high-risk patients without adjuvant chemotherapy. For ethical reasons, such studies are not possible today.

All patients included in the study received standard chemotherapy based on the physician's judgment and good clinical practice at the time of treatment and consequently, treatment regimens differ from those currently employed. The results, though, show an association between TIMP-1 and lack of benefit from anthracycline-based therapy, which is widely used in the adjuvant setting. A recent study showed a similar association between TIMP-1 and decreased benefit from anthracyclines [7], thus indicating that further validation of TIMP-1 as a predictive marker for anthracycline sensitivity is justified.

The exact mechanism by which TIMP-1 may affect the sensitivity to certain types of cytotoxic drugs has not been elucidated. However, the ability of TIMP-1 to influence cell growth and apoptosis is likely to play a role (reviewed in [4,5]). In keeping with this, *in vitro *preclinical findings from our laboratory show increased chemotherapy-induced apoptosis in cells made gene-deficient for the TIMP-1 gene when compared with the wild-type cells [26]. We recently extended the *in vitro *studies to a human breast carcinoma cell line and showed that cells expressing high levels of TIMP-1 protein were significantly less sensitive to treatment with etoposide and epirubicin than cells expressing low levels of TIMP-1 [27]. Thus, TIMP-1-mediated inhibition of chemotherapy-induced apoptosis could explain the decreased effectiveness of certain cytotoxic drugs in tumors producing large amounts of TIMP-1.

## Conclusion

This study suggests that patients with TIMP-1 high tumors benefit less from adjuvant chemotherapy with anthracyclines than patients with TIMP-1 low tumors. In the cohort studied here we were not able to find any, or only a very limited, prognostic impact of TIMP-1 and still, we observed a difference in outcome of TIMP-1 high and low patients after adjuvant anthracycline-based chemotherapy. We suggest that this difference is due to a predictive impact of TIMP-1. Despite the small size of the anthracycline-treated subgroup, the analysis of DFS clearly indicated an association between high levels of tumor tissue TIMP-1 and a shorter survival. Looking at OS, high levels of TIMP-1 were associated with a significantly shorter survival and we found a statistically significant interaction between TIMP-1 and anthracycline therapy. In the subgroup of CMF-treated patients, we found no consistent associations between TIMP-1 and survival. The results of this retrospective analysis are in keeping with other reports and our findings warrant further validation in prospective studies.

## Abbreviations

A: Adriamycin; CEF/CAF: Cyclophosphamide and 5-fluorouracil with epirubicin/adriamycin; CI: Confidence interval; CMF: Cyclophosphamide-methotrexate-5-fluorouracil; DFS: Disease-free survival; ELISA: Enzyme-linked immunosorbent assay; EORTC: European Organisation for Research and Treatment of Cancer; ER: Estrogen receptor; HR: Hazard ratio; MMP: Matrix metalloproteinase; OS: Overall survival; PgR: Progesterone receptor; RT: Radiotherapy; TIMP-1: Tissue inhibitor of metalloproteinases-1

## Competing interests

AS, MEMG, JAF and NB are co-applicants on the patent application "Cancer treatment and cancer treatment efficacy prediction by blocking and detecting protease inhibitors".

## Authors' contributions

AS wrote the paper and coordinated the analyses with MPL. MPL performed all statistical analyses with assistance from MEMG. MPL and MEMG administered the database with patient information. All authors participated in designing the study, in discussing content of the paper, and read and approved the final manuscript.

## Pre-publication history

The pre-publication history for this paper can be accessed here:

http://www.biomedcentral.com/1471-2407/9/322/prepub
